# Does Enhanced Recovery After Surgery Protocols Reduce Complications and Length of Stay After Thoracic Surgery: A Systematic Review of the Literature

**DOI:** 10.7759/cureus.59918

**Published:** 2024-05-08

**Authors:** Joshua G Goldblatt, Liam Bibo, Lachlan Crawford

**Affiliations:** 1 Cardiothoracic Surgery, Princess Alexandra Hospital, Brisbane, AUS; 2 Cardiothoracic Surgery, Fiona Stanley Hospital, Perth, AUS

**Keywords:** lung surgery, thoracic surgeries, general thoracic surgery, eras protocols, : enhanced recovery after surgery (eras)

## Abstract

Enhanced recovery after surgery (ERAS) has an increasingly important role in the perioperative management of thoracic surgical patients. It has been extensively studied in multiple surgical specialties, particularly colorectal surgery, where ERAS protocols have been shown to reduce postoperative length of stay and postoperative complications.

Electronic searches of two research databases were performed: PubMed (1972 to October 2023) and Ovid MEDLINE (1946 to October 2023). The literature search was completed on January 4, 2024. Search terms included: "thoracic surgery" and "ERAS" or "Enhanced Recovery After Surgery". The search was limited to studies evaluating humans undergoing thoracic surgery for any indication. The primary outcome was overall morbidity, with secondary outcomes including mortality, length of stay, and pulmonary complications.

The search yielded a total of 794 records, of which 30 (four meta-analyses and 26 observational trials) met the relevant inclusion and exclusion criteria. This review suggested the implementation of ERAS protocols can lead to a reduction in postoperative morbidity; however, this was not a consistent finding. The majority of studies included demonstrated a reduction in the length of stay with the implementation of ERAS.

Overall, ERAS/ERATS is an important adjunct to the management of patients requiring thoracic surgery, consistently leading to shorter lengths of stay and likely contributing to reduced rates of postoperative morbidity. Further research will be required to determine the impact of the recently released ERATS guidelines.

## Introduction and background

Enhanced recovery after surgery (ERAS) has an increasingly important role in the perioperative management of thoracic surgical patients. It has been extensively studied in multiple surgical specialities, particularly colorectal surgery, where ERAS protocols have been shown to reduce postoperative length of stay and postoperative complications [1-3]. ERAS pathways focus on optimising patient outcomes by concentrating on three key domains: preoperative optimisation, perioperative management, and postoperative management [1]. The principle is that the application of multiple, evidence-based interventions throughout the patient journey has a synergistic effect greater than the sum of all the parts.

The application of ERAS pathways in thoracic surgery has been shown to reduce postoperative length of stay in most studies and potentially reduce postoperative complications [4,5]. Furthermore, it has been demonstrated that improved compliance with each part of the ERAS protocol can lead to reduced postoperative morbidity [6]. In 2019, the ERAS Society and the European Society of Thoracic Surgeons (ESTS) published guidelines for ERAS in thoracic surgery, known as Enhanced Recovery after Thoracic Surgery (ERATS) [1]. These guidelines stipulated 45 ERAS outcomes across the three domains [1].

Since the publication of the 2019 guidelines, there has been limited research assessing the impact of ERATS programs on patient outcomes. The purpose of this review is to summarise the available literature and objectively appraise the influence of ERATS on patient outcomes.

Methods

This study was a systematic review completed according to the guidelines of the Preferred Reporting Items for Systematic Reviews and Meta-Analyses (PRISMA) statement. Institutional ethical approval was not required for this study.

Systematic search

Electronic searches of two research databases were performed: PubMed (1972 to October 2023) and Ovid MEDLINE (1946 to October 2023). The literature search was completed on January 4, 2024. Search terms included: "thoracic surgery" and "ERAS" or "Enhanced Recovery After Surgery". The search was limited to studies evaluating humans undergoing thoracic surgery for any indication. The search included only adults 18 years and older, excluding any studies involving paediatric patient cohorts. Duplicate records were initially excluded. Title-abstract screening was then performed to determine suitability for inclusion, prior to full-text manuscript review. Articles were independently reviewed by two authors (JG and LB) in parallel, with disagreements resolved by a third author (LC).

Inclusion and exclusion criteria

Studies examining the effect of implementing an ERAS or ERATS protocol on patients undergoing thoracic surgery were included. This included systematic reviews and meta-analyses, randomised-controlled trials (RCTs), and observational studies. Studies including patients under 18 years of age and studies involving animal subjects were excluded. Conference abstracts and non-English language manuscripts were excluded.

Outcomes

The primary outcome of interest for this systematic review was overall postoperative morbidity as an aggregate measure. Secondary outcome measures included postoperative length of stay, mortality, and the incidence of postoperative pulmonary complications. This review specifically aimed to examine the impact of ERAS or ERATS protocols on each of these factors.

Data analysis

In total, four meta-analyses and 26 observational trials satisfied the inclusion criteria [3-26]. These trials are summarised in Table 1. Each trial was assessed for adherence to the 2019 ERATS guidelines, and pertinent outcomes data were retrieved for comparison. These findings were subsequently synthesised and then presented in a narrative review format.

**Table 1:** Summary of the Included Studies ERATS: enhanced recovery after thoracic surgery; RCTs: randomised-controlled trials; NSCLC: non-small cell lung cancer; VATS: video-assisted thorascopic surgery; IV: intravenous; CI: confidence interval; ERAS: enhanced recovery after surgery; LOS: length of stay; DM: diabetes mellitus; BMI: body mass index; VTE: venous thromboembolism; PONV: post-operative nausea and vomiting; OR: odds ratio; RATS: robotic-assisted thoracic surgery; PVB: paravertebral block; RR: relative risk; MiV: minimally invasive; MDT: multi-disciplinary team; ESB: erector spinae block; COPD: chronic obstructive pulmonary disease; FEV1/FVC: forced expiratory volume in 1 second over forced vital capacity.

Study	Design of Study	Country	Population	Control	ERATS Intervention	Outcomes
Li et al., 2017 [5]	Systematic review and meta-analysis, including a total of 7 RCTs (5 English language, 2 Chinese).		A total of 486 patients enrolled between 2007 and 2016. 97.1% had been diagnosed with primary NSCLC. 67.1% underwent lobectomy, 16% underwent pneumonectomy and 16.9% underwent sublobar resection. 19.3% of the cohort underwent VATs.	Varied by study.	Varied between the studies, but the range was 5 to 11 interventions. These most commonly included (≥4 studies) the following: (1) preoperative: education and intensive pulmonary physiotherapy; (2) intraoperative: nil ≥ 4; (3) postoperative: epidural analgesia/NSAIDs, IV fluid restriction, early oral intake, and early ambulation	The primary outcome measure was overall morbidity, reported in 7/7 studies. The pooled relative risk for this was 0.64 (95% CI: 0.51-0.80), with a decreased overall morbidity with ERAS. No difference in mortality (reported in 4/7 studies). Regarding LOS, unable to complete due to heterogeneity (I^2^=86.5%). Reported in 7/7 studies, with mean LOS significantly shortened in the ERAS cohort in 4 of these studies. 3 studies showed no statistical difference. Reduced rates of pulmonary complications with a relative risk of 0.43 (95% CI 0.31-0.60).
Fiore et al., 2016 [4]	Systematic review and meta-analysis including 1 RCT and 5 non-RCT studies. Non-RCT all had a high risk of bias in the majority of domains of the Cochrane tool.		Included a total of 1612 patients, with 821 in the ERAS cohort and 791 in the control. 2 studies only included patients undergoing lobectomy. The remaining 4 included any pulmonary resection. The proportion of VATS was higher in the ERAS cohort.	Varied by study.	Described a total of 15 elements, with the number used ranging from 4 to 10, a median of 6.5. The most frequently cited included: (1) preoperative: education (n=5) and prophylactic antibiotics (n=4); (2) intraoperative: epidural (n=4); (3) postoperative: standard chest tube management (n=5) and early mobilisation (n=4)	Results included: (1) LOS: significantly reduced in the non-RCT (difference 1.2-9.1 days), with no difference in the RCT; (2) Readmission rates: Variably reported (3 non-RCT) with rates 1-10%. Note that 1 study spanned 7 days, 1 study spanned 30 days, and no time period was specified in 1 study; (3) Complications Reported in 3/6 studies. No difference in the overall complication rate. RCT showed a significant reduction in pulmonary complications with ERAS; (4) Mortality reported in 3/6 studies with no difference.
Li et al., 2021 [7]	Systematic review and meta-analysis included 21 articles in total: 19 cohort studies and 2 RCTs. The range was 2006 to 2020.		6,480 patients total, with 2,617 patients in ERAS cohorts and 3,863 patients in the control.		The number utilised in the ERAS group ranged from 5 to 22 and that in the non-ERAS cohort ranged from 0 to 10. The most used included: preadmission education (19/21 studies), early ambulation (18/21 studies), VATS (12/21 studies), rational use of analgesics postop (17/21 studies), standardised chest tube management (15/21 studies), early oral intake postop (12/21 studies), and standard anaesthetic protocol (13/21 studies)	Overall complication rate was reported in 18 studies. There was a reduced incidence of postoperative complications in the ERAS cohort with a pooled RR 0.64 (95% CI 0.52-0.78, p<0.001). There was significant heterogeneity (I^2^=63%). In-hospital mortality was reported in 13 studies, with all studies reporting no difference. The LOS was divided into overall LOS (11 studies) and postoperative LOS (12 studies). Both were reduced in the ERAS cohort in most studies. Pooled analysis was only available for 7 studies with ERAS patients having significantly shorter LOS (-1.58, 95% CI: -2.38 to -0.79). There was significant heterogeneity (I^2^=98%). Pulmonary complications were lower with ERAS, RR 0.58, 95% CI 0.45-0.75, p<0.001.
Zhang et al., 2021 [8]	Systematic review and meta-analysis including 23 studies: 12 cohort studies and 11 RCTs. Included all patients undergoing surgery for lung cancer in which there was at least 1 ERAS protocol implemented.		8,094 patients were included in total, with 3,151 in the ERAS cohort and 4,943 in the control group.	Varied by study.	Each study used different ERAS measures. The specific of each study is outlined.	22/23 studies reported on overall complications. ERAS significantly reduced the incidence of complications, OR 0.48 (95% CI 0.37-0.61, p<0.01). There was significant heterogeneity, I^2^=62%. The event rate of mortality was low. There was no difference between the groups (OR 1.15, 95% CI 0.6-2.22, p>0.05). 20/23 studies reported on LOS, with a reduction in the ERAS cohort of -2.70 (p<0.01). Once again, significant heterogeneity, I^2^=97%.
Alwartari et al., 2021 [13]	Retrospective cohort study looking at three time periods between 2005 and 2019. The first was pre-ERAS (2005-11), the second transitional period (2012-15) and the third was ERAS (2016-19). All patients underwent open lobectomy in this study.		10,021 patients in total. 49% male, mean age 67 years old. The 2016-19 cohort had higher rates of DM, BMI, and ASA classifications.	Control included the transitional period (2012-15) and the pre-ERAS period (2005-11).	No comment on the specific ERATS interventions in the 2016-19 period.	Demonstrated that patients in the 2016-19 compared to the previous periods had: lower rates of unplanned reintubation; lower rates of surgical site infections; lower mortality (1.9% vs 2% vs 2.8%, p=0.05); and shorter LOS (6.6 days vs 7.1 days vs 8.1 days, p<0.01). There was no difference in rates of pneumonia, reoperation, or readmission across the time periods.
Thompson et al., 2021 [14]	Prospective, longitudinal study between 2017 and 2019. There are three phases including 9 months pre-ERATS, 3 months implementation periods and 9 months post-ERATS implementation.		704 patients in total were included, with 352 patients included in each cohort (pre- and post-ERATS). Included all patients undergoing thoracic surgery: lung resection, oesophagectomy, gastrectomy and paraoesophageal hernia repair. No major difference in baseline characteristics. 67% of procedures were minimally invasive lobectomies or sublobar resections.	Included a pre-ERATS period, which is the initial 9 months of this study.	(1) Preoperative: Patient education; (2) Intraoperative: Standardised anaesthesia plan and pathway, perioperative multimodal analgesia, and reversal of muscle relaxant; and (3) Postoperative: Standard order sets Multimodal analgesia Early ambulation Early chest tube removal Early removal of urinary catheter Early oral feeding	There was no mortality data in this study. The overall LOS was shorter in the post-ERATS cohort: 4.7 days vs 6.2 days, p=0.011. This was driven by patients who underwent open oesophagectomy, open lobectomy and sublobar resections. No difference in overall major or minor complications. The authors demonstrated an improved 6-minute walk test in the post-ERATS cohort: 402 metres vs 371 metres, p=0.046.
Rogers et al., 2018 [6]	Prospective cohort study including all patients undergoing lung resection for primary lung cancer between 2012 and 2014 at a single institution.		422 patients included. There is no historical comparison group in this study. 71.6% underwent VATS. 70.4% underwent lobectomy.	There was no control group in this study; however, compliance with the ERAS protocol was used as a surrogate measure.	15-element ERAS protocol, with additional specific components for thoracic surgical patients. Preoperative included: education, assessment, smoking cessation, prehabilitation, day of surgery admission, preoperative carbohydrate drink, and avoidance of sedative premedications. Intraoperative included: prophylactic antibiotics, regional anaesthesia with paravertebral, avoidance of too much crystalloid, intraoperative warming, VTE prophylaxis, no urinary catheter, VATS, when possible, and single chest drain. Postoperative: avoidance of IV fluids and opioids, early feeding, targeted PONV treatment, early mobilisation within 24 hours, early chest drain removal	The median LOS was 5 days. Improved compliance with the ERAS protocol was associated with an inverse relationship with morbidity: OR 0.72, 95% CI 0.57-0.91.
Han et al., 2022 [15]	Retrospective cohort study including all patients undergoing VATS or RATS lobectomy at a single institution. Pre-ERAS (October 2018 to September 2019) and post-ERAS (October 2019 to September 2020).		116 patients were included. RATS approach 51%. No difference between RATS and VATS cohorts at baseline. ERAS pathway in 55%. No difference between pre- or post-ERAS cohorts.	The study was twofold: comparing RATS to VATS and comparing the effect of ERAS.	Detailed ERAS protocol documented in the study. Preoperative: education, no prolonged fasting, smoking cessation, same-day admission. Perioperative: avoid hypothermia, VTE prophylaxis, antibiotics, no urinary catheter, standardised anaesthetic technique, PVB Postoperative: VTE prophylaxis, chest drain management protocol, digital chest drains, early oral intake, PONV prevention, multimodal oral analgesia, early mobilisation.	ERAS decreased the LOS by 1.23 days. There was no difference in major post-operative complications or 30-day readmission. Mortality not reported.
Haro et al., 2021 [9]	Propensity-matched prospective cohort study including patients who underwent elective lobectomy or sublobar resection for primary lung cancer or pulmonary metastasis. The two cohorts included pre-ERAS from 2015 to 2017 and post-ERAS from 2017 to 2019.		295 patients included. There were 169 patients pre-ERAS and 126 patients post-ERAS. No difference in baseline characteristics. 40% MiV pre- and 63% post-ERAS.	In this group, the patient pathway was not standardised. All patients received epidurals.	The ERAS program included: Preoperative: education, a surgery to-do list. Intraoperative: protocolled analgesia, epidural for only thoracotomy, normothermia, prophylaxis (antibiotics, VTE), lung-protective ventilation, goal-directed euvolaemia. Postoperative: standardised LOS, multi-modal analgesia, early chest drain removal, early diet, early ambulation.	After propensity-matching, there was an absolute reduction in overall morbidity from the implementation of ERAS of 13.6% (p=0.02). The LOS was reduced by 1.4 days (p<0.01) from 4.5 to 3.1 days. When accounting for the MIV approach, the reduction in LOS and overall morbidity remained with the implementation of ERAS.
Wang et al., 2021 [16]	Retrospective cohort study looking at implementation of ERAS at a single institution. Two time periods: routine 2012-2014 and ERAS 2016-2017. All patients undergoing anatomical resection for NSCLC.	China	1,749 patients were included, with 691 in the ERAS group and 1,058 in the routine group. Higher rate VATS in ERAS cohort (74.2% vs 65.3%, p<0.001).	Pathway of management is well described. No standardised preoperative education or assessment. No standardised VTE prophylaxis. Routine urinary catheter. Non-standardised chest drain removal. No standardised post-operative analgesia management.	Incorporated majority of original ERAS elements. Preoperative: education, consultation, smoking cessation, alcohol cessation. Perioperative: VTE prophylaxis, multi-modal analgesia. Postoperative: early mobilisation, no urinary catheter, early chest drain removal.	The ERAS cohort had a shorter postoperative LOS (4 vs 6 days, p<0.001) and shorter overall LOS (10 vs 13 days, p<0.001). ERAS cohort had lower rates of postoperative pulmonary complications: 15.2% vs 19.5%, p=0.022. This was driven by a reduction in rates of pneumonia (8.4% vs 14.2%, p<0.001) and atelectasis (5.9% vs 9.8%, p=0.004). There was no difference in rates of prolonged air leaks or mortality.
Martin et al., 2018 [17]	Prospective cohort study including historical control from the pre-ERAS era. Looked at two pathways: VATS and open, and the impact of ERAS on each. Included all elective patients undergoing either VATS or thoracotomy.	USA	363 patients in total divided into four cohorts: ERAS VATS (n=81), control-VATS (n=162), ERAS thoracotomy (n=58), and control-thoracotomy (n=62). No difference in balance characteristics across the cohorts.	Historical controls from the pre-ERAS era within the year 2015 (ERAS implemented in 2016).	Developed own protocol. Of note, the highly standardised approach to intraoperative and postoperative pain management. Preoperative: education. Perioperative: preoperative drink, regional anaesthesia, standardised multimodal analgesia, VTE prophylaxis, antibiotics, lung-protective ventilation. Postoperative: standardised chest drain management, multi-modal analgesia, early diet, early urinary catheter removal (note this was changed due to high rates of urinary retention with no urinary catheter in the initial phase of protocol)	Median LOS reduced in both ERAS VATS (2 days vs 3 days, p=0.07) and ERAS thoracotomy (4 days vs 6 days, p=0.009). No difference in complication rates between ERAS VATS and historical controls. The ERAS thoracotomy cohort had similar rates of complications to historical control other than atelectasis requiring bronchoscopy (3% vs 14%, p=0.05).
Bellas-Cotan et al., 2021 [18]	Cohort study, with prospective ERAS cohort and retrospective pre-ERAS controls.	Brazil	100 patients total, 50 in ERAS (2018-19) and 50 in historical controls (2016). Of note, significantly lower rates of COPD in the ERAS cohort (8% vs 24%, p=0.03) and lower rates of ASA>2 (30% vs 52%, p=0.03). VATS 52% in ERAS vs 22% standard (p<0.001).	Historical controls drawn from 2016 which was prior to the ERAS era.	Own ERAS protocol. Preoperative: MDT education, smoking cessation, nutritional screening. Perioperative: VATS preferred, VTE prophylaxis, antibiotics, minimised fasting, regional anaesthesia (epidural, ESB or intercostal blocks), normothermia. Postoperative: early oral intake, and early ambulation.	No difference in surgical complications (ERAS 24% vs standard 36%, p=0.19), or non-surgical complications (ERAS 24% vs standard 42%, p=0.06). There was no difference in LOS (4 vs 4 days, p=0.19). No difference in mortality, but 0 deaths in ERAS vs 2 deaths in standard. Multi-variate analysis showed ERAS adherence associated with lower incidence surgical complications.
Shiono et al., 2018 [19]	Cohort study with prospective ERAS cohort (2015-2018) and historical cohort as control (2013-2015). Included all patients undergoing complete resection for lung cancer or pulmonary metastasis. Also divided into three groups based on age: <65, 65-74 and ≥75 years old.	Japan	535 patients were included after exclusion criteria were applied. This included 130 in the ERAS cohort and 405 in the standard cohort. All patients underwent thoracotomy. Propensity-matching was performed with 130 patients in each arm.	Historical controls from 2013-15 pre-ERAS. Management was at the discretion of the treating physician. The preference was for epidural anaesthesia.	Own ERAS protocol. Preoperative: education, chest rehabilitation. Intraoperative: PVB. Postoperative: early ambulation, early oral intake, standardised chest drain removal, multimodal analgesia.	Prior to propensity-matching, the ERAS cohort had shorter postoperative LOS and a lower incidence of morbidity. There was no difference in mortality. Pulmonary complications occurred in 5.4% ERAS vs 10.6% control (p=0.06). After propensity-matching, ERAS had shorter postoperative LOS (4 vs 5 days, p<0.001). There was no difference in overall morbidity (12.7% vs 19.1%, p=0.167). There was no difference in mortality.
Tiberi et al., 2021 [20]	A retrospective study looking at patients who underwent uniportal VATS in a pre- and post-ERAS era. Included all lung resections: lobectomy, segmentectomy and wedge for benign or malignant indications.	Italy	1,167 patients were included in total, with 985 in the control group (2015-2019) and 182 in the ERAS group (2019-2020). Propensity-matching was subsequently performed. There were statistically significant differences in baseline, but not clinically meaningful (i.e. age 66.2 years ERAS vs 68.1 control, p=0.03).	History control. There is no mention of the standard of care prior to the implementation of ERAS.	Own ERAS protocol. Preoperative: MDT education, smoking cessation, nutritional screening, and treatment, prehabilitation. Perioperative: antibiotics, VTE prophylaxis, normothermia maintenance, standardised anaesthesia, multimodal analgesia, PVB or ESB. Postoperative: early mobilisation, early oral intake, multimodal analgesia, standard chest drain management.	Results were reported after propensity-matching. ERAS was associated with shorter mean LOS by 1 day (3.13 vs 4.18 days, p<0.0001). There was no difference in cardiorespiratory complications (12.1% ERAS vs 11% control, p=0.74).
Forster et al., 2020 [12]	Retrospective cohort study looking at the impact of compliance with ERAS protocols. Included all patients undergoing anatomic lung resection by VATS. High compliance was defined as >75% adherence to the protocol, and low compliance as ≤75% adherence.	Switzerland	192 patients in total with 93 in the high compliance and 99 in the low compliance cohorts. 57% underwent lobectomy and 43% segmentectomy. All were via VATS. Overall compliance with ERAS protocols was 76%.	Defined as compliance ≤75% with the ERAS protocol.	Adapted from the ERAS/ESTS guidelines published in 2013. Preoperative: education. Perioperative: carbohydrate drink, avoidance of sedatives, VTE prophylaxis, antibiotics, normothermia, maintenance euvolaemia, no urinary catheter, multimodal analgesia, no epidural, PONV prophylaxis. Postoperative: standardised chest drain management, early mobilisation, multimodal analgesia.	Patients in the high compliance group had lower rates of complications (18% vs 48%, p<0.001). This was driven by lower rates of pulmonary complications (16% vs 38%, p=0.005). There was no difference in the LOS (4 vs 5 days, p=0.17). There were no mortalities.
Forster et al., 2020 [21]	Retrospective cohort study looking at patients undergoing VATS lobectomy for NSCLC between 2014 and 2019. Two groups: pre- and post-ERAS. Same unit as Forster et al.	Switzerland	307 patients total, with 167 pre- and 140 post-ERAS. No major difference in baseline characteristics. Overall compliance with the ERAS protocol was 81%. Propensity-matching was also performed.	Prior to the implementation of the ERAS protocol. Of note, preference was for epidural anaesthesia or intercostal nerve blocks. Not all care pathways were standardised, but there was standardised chest drain management.	The same as documented by Forster et al., as this is the same institution during a similar study period.	The unadjusted analysis demonstrated that implementation of ERAS led to reduced LOS: 5 days vs 7 days (p=0.04). This difference remained significant after propensity-matching. Overall morbidity was no different in both unadjusted and propensity-matched analyses. Propensity-matching demonstrated a 13% reduction in cardiopulmonary complications in the ERAS cohort. No comment on mortality.
Young et al., 2024 [22]	Prospective cohort study looking at all patients undergoing elective pleural, pulmonary, or mediastinal surgery at a single institution between 2015 and 2021. ERAS protocol was implemented in 2016, giving pre-ERAS and post-ERAS cohorts. Additional division according by approach: VATS or thoracotomy.	USA	1,079 patients in total, classified into four cohorts: pre-ERAS VATS (n=164), post-ERAS VATS (n=600), pre-ERAS thoracotomy (n=60), post-ERAS thoracotomy (n=255). No major difference in baseline characteristics.	Controls were the pre-ERAS group, with each divided by approach: VATS or thoracotomy. Pre-ERAS care was at the discretion of the physician.	Protocol devised within this institution is based on ERAS principles. Multiple adjustments were made over the study period including AF prophylaxis, adjustment to gabapentin dosage and post-discharge nursing phone call. Preoperative: education. Perioperative: minimal fasting, avoidance of pre-medication sedatives, multi-modal analgesia with regional techniques, protective lung ventilation. Postoperative: multimodal analgesia, early mobilisation, standardised chest drain management.	No difference in mortality (only 1 mortality in this study). There was no overall morbidity rate reported. LOS was reduced in both ERAS cohorts. In the thoracotomy cohort 4 days vs 5.5 days, p=0.01 and in VATS cohort 2 vs 3 days, p<0.01. Multivariate analysis found year of surgery and VATS were associated with a reduction in atelectasis requiring bronchoscopy (OR 0.83, p=0.048), AF (OR 0.74, p<0.001) and urinary retention (OR 0.84, p<0.001).
Lee et al., 2021 [3]	Prospective longitudinal study over 21 months 2017 to 2019. This spanned the introduction of the ERATS program. There were three periods: 9 months prior to ERATS, 3-month transition phase and 9 months post-ERATS. The post-ERATS cohort was further divided into 3, 3-monthly cohorts. The 3-month transition phase was excluded from the study. All patients undergoing elective major thoracic surgery.	Canada	704 patients were included in total, with 352 patients in each of the pre- and post-ERATS cohorts. The majority underwent VATS lobectomy or sublobar resection.	9-month period to the implementation of the ERATS protocol. The 3-month transition period was excluded.	ERATS protocol instituted over a 3-month period. Included: Preoperative: education, smoking cessation, prehabilitation. Perioperative: standardised anaesthetic protocols. Postoperative: early ambulation, early diet, standardised chest drain management.	There was no change in the median LOS (3 days) across the study period. However, over the study period, there was a reduction in LOS for those undergoing VATS lobectomy (4.3 to 3.3 days, p=0.34) and VATS sublobar resection (3.4 to 2.2 days, p=0.13). The incidence of minor complications decreased from 18.2% in the pre-ERATS to 7.9% in the 7–9-month post-ERATS cohort (p=0.009). Major complications also decreased from 13.6% pre-ERATS to 4.4% in the 7–9-month post-ERATS cohort (p=0.007).
Brunelli et al., 2017 [23]	Retrospective cohort study on prospectively collected data between 2014 and 2017. Included all patients undergoing VATS lobectomy (n=561) or VATS anatomic segmentectomy (n=39).	England	600 patients were included in total, with 365 patients pre-ERAS and 235 post-ERAS. There was no major difference in baseline characteristics.	Control was pre-adoption of formal ERAS protocol. Although, a lot of elements are already being practised. Including multimodal analgesia, early mobilisation, standardised chest drain management and intraoperative regional anaesthesia.	ERAS protocol was implemented in 2016 which formalised the processes. Previous processes continued. Preoperative: education, prehabilitation. Perioperative: minimal fasting, normothermia. Postoperative: early mobilisation, early oral diet, PONV prevention, preventing fluid overload, multimodal analgesia.	There was no difference in LOS in the two cohorts (pre-ERAS 5 days vs post-ERAS 4 days, p=0.44). There was no difference in in-hospital mortality between the two cohorts (pre-ERAS 2.2% vs post-ERAS 3.8%, p=0.31). There was also no difference in 90-day mortality: pre-ERAS 3% vs post-ERAS 4.7%, p=0.34. No difference in the incidence of cardiovascular and pulmonary complications (pre-ERAS 22.6% vs post-ERAS 22.4%, p=0.98).
Scarci et al., 2015 [24]	Retrospective cohort study including pre- and post-implementation of ERAS.	England	325 patients were included, with 154 patients in the ERAS cohort and 171 historical controls. No mention of baseline characteristics between cohorts.	Historical controls prior to implementation of ERAS protocol. No mention of pre-ERAS management pathways.	Hospital-based ERAS protocol developed from the best available evidence. Preoperative: MDT education, smoking cessation, nutrition management, prehabilitation, correction of anaemia. Perioperative: minimise fasting, minimise premedications, antibiotics, DVT prophylaxis, multi-modal analgesia, VATS, and avoid urinary catheters. Postoperative: PONV prophylaxis, multimodal analgesia, standard chest drain management, early mobilisation.	LOS only outcome which was reduced in the ERAS cohort: 5.2 days vs 11.7 days, p<0.0001.
Tahiri et al, 2019 [25]	Prospective cohort study, using historical controls. Propensity-matching was also performed. Included all patients undergoing VATS lobectomy, with those being converted to open excluded.	Canada	98 patients were included in each cohort. No major difference in the baseline characteristics. The adherence to the ERAS protocol was 64.3%.	98 historical patients who underwent VATS lobectomy by the same surgeons in the previous year.	Hospital-derived ERAS protocol. No comment on the preoperative interventions. Perioperative: intercostal nerve block favoured over epidural, although epidural could still be used. Postoperative: standardised chest drain management, minimise urinary catheter use, early diet, early ambulation	Median LOS was reduced with ERAS, 5 vs 3 days, p<0.001. There was no difference in the overall morbidity rate, with 23.4% in the ERAS group vs 28.6% in the control.
Huang et al., 2017 [26]	Retrospective cohort study at a single institution between 2016 and 2017. All patients were undergoing surgery for early-stage NSCLC.	China	83 patients total, with 38 in the ERAS cohort and 45 synchronous controls. No difference in the baseline characteristics. All underwent VATS.	45 patients, same study period, who did not receive the ERAS protocol. Conventional VATS approach.	Hospital-based ERAS protocol, Preoperative: education, smoking cessation, alcohol cessation, nutrition assessment and prehabilitation. Perioperative: VTE prophylaxis, minimise fasting, no sedative premedication, antibiotics, multimodal analgesia, regional anaesthesia, normothermia and euvolaemia. Postoperative: PONV prevention, standardised urinary catheter management and standardised chest drain management.	There were no in-hospital mortalities. The ERAS cohort had shorter LOS: 6.58 vs 8.69 days, p=0.024. There was no difference in the incidence of pulmonary complications (with a very low event rate). There was no difference in complications between the two cohorts.
Peng et al., 2021 [27]	Retrospective cohort study between 2017 and 2019. ERAS introduced the mid-point of the study (April 2018), which split into two cohorts. Also looked at the effect of VATS vs open approach.	USA	131 total patients were included with 64 pre-ERAS and 67 post-ERAS. No difference in baseline characteristics. 71.9% MIV approach pre-ERAS vs 59.7% post-ERAS era (p=0.07).	64 patients included.	Hospital-instituted ERAS protocol. Preoperative: education, anaemia management, prehabilitation. Perioperative: no sedative premedications, minimised fasting time, multi-modal opioid-sparing analgesia, regional anaesthesia with intercostal blocks, anaesthetic protocols, euvolaemia. No comment on the postoperative pathway.	There was no difference in postoperative LOS between the two cohorts. There were no in-hospital mortalities. There was no difference in the incidence of postoperative major complications: ERAS cohort 3% vs control cohort 4.7%, p=0.68. When stratified by approach, ERAS had no impact on postoperative LOS.
Chen et al. 2020 [28]	Retrospective cohort study at a single institution between 2015 and 2017. ERAS was introduced in September 2016, divided into two cohorts. All patients with resectable lung cancer, stage I to III.	China	337 patients in total, with 168 in control and 169 in ERAS cohort. No major difference in the baseline characteristics.	168 patients. Standard of care included admission education, early postoperative ambulation, and opioid-based analgesia.	Hospital-based protocol. Preoperative: education, prehabilitation. Postoperative: early ambulation. No comment on intraoperative interventions.	ERAS was associated with a reduced postoperative LOS: 8.91 vs 11.98 days, p<0.001. ERAS was associated with reduced postoperative lung infections: 2.96% vs 8.92%, p=0.020. There was no mortality data.
Abrao et al., 2021 [29]	Prospective cohort study using historical cohort as control. Patients requiring elective pulmonary resections. Patients underwent either open or VATS as the approach.	Brazil	122 patients were included in total, with 61 in the ERAS cohort and 61 in the control cohort. No major differences in baseline characteristics. Matched patients on surgical procedures and diagnoses. Predominant approach was thoracotomy, with 18% VATS in ERAS and 13.1% VATS in control.	61 patients in the control group who received conventional care.	Hospital-based protocol adapted from ERAS/ESTS. Preoperative: education, smoking cessation, prehabilitation Perioperative: minimise sedating pre-medications, minimise fasting, anaesthetic protocol, regional anaesthesia with ESB, antibiotics, Postoperative: early mobilisation, standardised chest drain management, multimodal analgesia.	ERAS group had reduced median LOS: 1 day vs 3 days, p<0.001. There was no difference in the incidence of complications (8.2% ERAS vs 9.8% control, p=0.752) or mortality (0 ERAS vs 1.6% control, p=1).
Laohathai et al., 2023 [30]	Retrospective cohort study between 2016 and 2020, with ERAS introduced in 2019. Propensity-matching (1:2) was used. All patients underwent elective pulmonary resection at a single institution.	Thailand	321 patients total, of which 247 were in the ERAS cohort, and 74 were controls, prior to ERAS. Prior to matching, the control group was older: 66.9 years vs 60 years, p<0.001. The proportion of those undergoing lobectomy was also higher in the control group: 54.1% vs 36.1%, p=0.002. VATS was also significantly more likely in the ERAS cohort: 93.5% vs 16.2%, p<0.001.	74 patients prior to ERAS. Patients received minimal pre-operative education including no smoking cessation advice. They received no regional anaesthesia.	Adapted from the ERAS guidelines. Preoperative: education, smoking cessation, correction of anaemia. Intraoperative: no sedative pre-medications, minimisation of fasting time, multimodal analgesia with intercostal nerve blocks. Postoperative: early mobilisation, multimodal analgesia, standardised chest drain management.	Prior to matching, the ERAS group had a significantly shorter LOS: 11 vs 6 days, p<0.001. This difference persisted after matching: 5 vs 7 days, p<0.001. There was no significant difference in the incidence of complications or mortality, including after matching, although the overall event rate was low.
Ni et al., 2021 [10]	Retrospective cohort study from 2018 to 2020 at a single institution undergoing VATS lobectomy. Two cohorts: ERAS (called ‘rapid recovery group’) and standard of care.	China	629 patients in total. The ERAS cohort was further divided into non-early discharge group (>72 hours, GROUP B, n=103) or early discharge group (≤72 hours, GROUP C, n=405). The control group, with the standard of care was GROUP A. There were no major differences in baseline characteristics.	Denoted as GROUP A, with 121 patients total.	‘Rapid recovery group’, with protocol adapted from ERAS. Preoperative: education (MDT-based), prehabilitation, nutritional screening Perioperative: minimisation of fasting, normothermia, regional anaesthesia and multimodal analgesia, standardised chest drain management, avoiding urinary catheters. Postoperative: early mobilisation.	Postoperative LOS was reduced with ERAS (Group A) compared to standard care: 2.74 vs 5.7 days, p<0.05. ERAS was also associated with a significant reduction in overall postoperative complications: 13.6% vs 38.8%, p<0.05. Mortality data were not reported on. Postoperative pulmonary complications were lower with ERAS: 8.5% vs 29.7%, p<0.05.
Turna et al., 2023 [11]	Single-centre retrospective centre between 2001 and 2021. Three groups: pre-ERAS 2001-10, preparation for ERAS 2011-15 and post-ERAS 2016-21. Included all patients receiving an operation for NSCLC.	Turkey	845 patients included in total. Three groups: pre-ERAS (Group 1, n=285), preparation for ERAS (Group 2, n=269) and post-ERAS (Group 3, n=291). Group 3 had lower rates of preoperative smoking and higher FEV1/FVC.	Group 1 was control, with 285 patients. No preoperative education and low uptake of VATS. Group 2, had minimal preoperative education, increased frequency of VATS and ongoing opioid-heavy analgesia regimes.	Based on the ERAS protocols. Preoperative: education, smoking cessation, pulmonary rehabilitation, nutritional assessment Perioperative: antibiotics, analgesia with epidural OR intercostal, euvolaemia, VTE prophylaxis, PONV prevention Postoperative: early mobilisation, early diet, multi-modal analgesia.	Group 3 (i.e. with ERAS) was associated with significantly shorter postoperative LOS: 5.5 vs 7 vs 8.5 days, p<0.001. Group 3 had a lower incidence of overall complications (no specific values, but p<0.001).
Madani et al., 2015 [31]	Single-centre retrospective cohort study between 2011 and 2013. From 2012, patients underwent treatment under an ERAS protocol. Included all patients undergoing an open lobectomy for primary or secondary cancer. Notably, all patients who underwent VATS were excluded.	Canada	234 patients met the study inclusion criteria for a total of 448 patients. 127 patients were in the pre-ERAS group and 107 in the post-ERAS group. No major differences in baseline demographics.	The control group received the standard of care and comprised 127 patients.	Hospital developed protocol, based on ERAS principles. Preoperative: education. Perioperative: preference for epidural anaesthesia Postoperative: early mobilisation, standardised chest drain management.	LOS was significantly reduced with ERAS: 6 vs 7 days (p<0.01). The incidence of overall morbidity was reduced in the ERAS cohort: 37% vs 50%, p=0.03. This difference however was driven by a reduction in urinary tract infections (3% vs 12%, p<0.01). There was a difference in the incidence of any pulmonary complications: 25% vs 31%, p=0.38. There was no difference in mortality, with only 1 mortality in the entire cohort.
Van Haren et al., 2018 [32]	Single-centre retrospective cohort study between 2006 and 2016. Divided into three cohorts: pre-ERAS (2006-11), transitional period (2012-15) and post-ERAS (2015-2016). Included all patients undergoing anatomic and non-anatomic resection for primary lung cancer.	USA	2,886 patients, with 1,615 in the pre-ERAS cohort, 929 in the transitional cohort and 342 in the ERAS cohort. Interestingly, there was an improvement in preoperative ECOG scores from pre-ERAS to transitional to ERAS. The proportion of MiV surgery also increased across the study period, with 32.9% VATS/RATS in the pre-ERAS cohort compared to 46.8% in the transitional compared to 51.1% in the ERAS cohort (p<0.001).	Standard of care included prophylactic antibiotics and VTE prophylaxis.	ERAS protocol developed within the hospital. Preoperative: education. Perioperative: minimise fasting time, antibiotics, VTE prophylaxis, PONV prevention, minimise sedative pre-medications, standard anaesthetic protocol, euvolaemia Postoperative: early ambulation, early diet, standardised chest drain management	LOS reduced across the time of the study: 5 days (pre-ERAS) vs 4 days (transitional) vs 4 days (ERAS), p<0.001. There was no difference in overall mortality across the study period: 1% vs 0.5% vs 0.6%, p=0.417. There was no comment on the overall complication rate. The authors found that ERAS patients had lower rates of pulmonary complications (19.9% vs 28.7%, p=0.004) and cardiac complications (12.3% vs 18.1%, p=0.001). On subgroup analysis, this difference in pulmonary and cardiac complications was only true for patients who underwent thoracotomy, and not MiV.

## Review

Results

**Figure 1:**
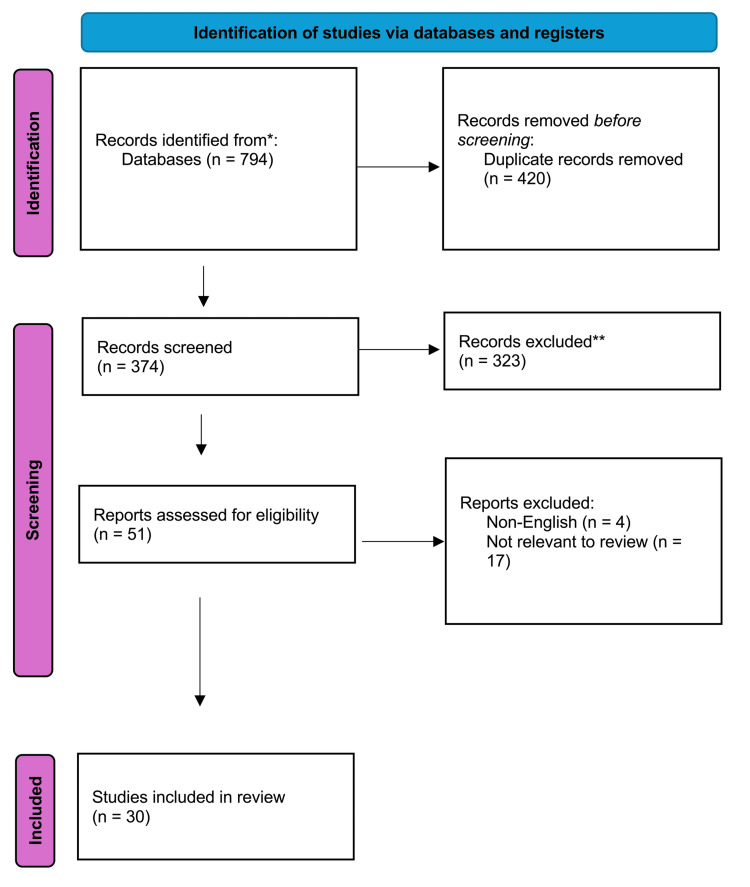
Preferred Reporting Items for Systematic Reviews and Meta-Analyses (PRISMA) flowchart.

The initial search yielded 794 records; after removal of duplicates, 374 records were suitable for screening. The titles and abstracts of these articles were reviewed by two independent authors (JG and LB), with 323 records excluded as they did not fit the inclusion criteria. A total of 51 records were retrieved for review of the full text. After full-text review, a further 21 records were excluded for a final included total of 30 publications, of which four were meta-analyses and 26 were observational cohort studies [10-16]. The PRISMA diagram is shown in Figure 1. 

Extent and rigour of ERATS protocol implementation

The breadth of ERAS implementation and the degree of protocol compliance varies significantly among all the studies, including those evaluated within each meta-analysis. Table 2 depicts the components of the 2019 ERATS protocol that each of the included observational studies utilised, categorised under 21 major groups, noting that there are 45 individual components of the ERATS protocol [1]. It is important to note that most of the studies either pre-date the release of the 2019 ERATS guidelines or include cohorts of patients which are pre-2019. The range of components implemented was 4-18 with a median of 10.5. The most frequently implemented sections of the protocol were pre-operative education and early mobilisation post-surgery, with 24 of the 26 studies implementing both strategies. The least implemented components were atrial fibrillation prevention (one of 26 studies) and documented alcohol dependency management plan (2 of 26 studies).

**Table 2:** ERAS/ERATS Protocols Utilised in Each Study

	Alwatari et al. [13]	Thompson et al. [14]	Rogers et al. [6]	Wang et al. [16]	Martin et al. [17]	Bellas-Cotin et al. [18]	Shiono et al. [19]	Tiberi et al. [20]	Forster et al. [12]	Forster et al. [21]	Young et al. [22]	Lee et al. [3]
Preoperative												
Preadmission information, counselling, and education	No comment on any ERAS protocol used	√	√	√	√	√	√	√	√	√	√	√
Perioperative nutrition				√		√		√				
Smoking cessation			√	√		√	√	√	√	√		
Alcohol dependency management				√								
Anaemia management												
Pulmonary prehabilitation			√					√				
Admission												
Preoperative fasting and carbohydrate treatment			√		√	√			√	√	√	
Preanaesthetic medication		√	√						√	√	√	
Perioperative phase												
VTE prophylaxis			√	√	√	√		√	√	√		
Antibiotic prophylaxis			√			√	√	√	√	√		
Avoiding hypothermia			√			√		√	√	√		
Standard anaesthetic protocol		√	√		√			√	√	√	√	√
PONV control			√				√		√	√		
Regional anaesthesia and analgesia		√	√	√	√	√		√	√	√	√	
Perioperative fluid management			√		√	√			√	√	√	
AF prevention											√	
Surgical technique: thoracotomy							√				√	
Surgical technique: MIV			√			√		√	√	√	√	
Postoperative phase												
Chest drain management		√	√	√	√		√	√	√	√	√	√
Urinary drainage			√	√	√			√	√	√		
Early mobilisation		√	√	√	√	√	√	√	√	√	√	√

	Brunelli et al. [23]	Scarci et al. [24]	Tahiri et al. [25]	Huang et al. [26]	Peng et al. [27]	Chen et al. [28]	Abrão et al. [29]	Laohathai et al. [30]	Ni et al. [10]	Turna et al. [11]	Madani et al. [31]	Van Haren et al. [33]	Han et al. [15]	Haro et al. [9]
Preoperative														
Preadmission information, counselling, and education	√	√		√	√	√	√	√	√	√	√	√	√	√
Perioperative nutrition		√		√					√	√				
Smoking cessation		√		√			√	√		√			√	
Alcohol dependency management				√										
Anaemia management		√			√			√						
Pulmonary prehabilitation	√	√		√	√		√		√	√				
Admission														
Preoperative fasting and carbohydrate treatment	√	√		√	√		√	√	√			√	√	
Preanaesthetic medication		√		√	√		√	√				√		
Perioperative phase														
VTE prophylaxis	√	√		√						√		√	√	√
Antibiotic prophylaxis	√	√		√			√	√	√	√		√	√	√
Avoiding hypothermia	√			√					√				√	√
Standard anaesthetic protocol	√	√		√	√		√		√			√	√	√
PONV control	√	√		√					√	√		√	√	
Regional anaesthesia and analgesia	√	√	√	√	√		√	√	√			√	√	√
Perioperative fluid management	√	√		√	√			√	√	√		√		√
AF prevention														
Surgical technique: thoracotomy								√						
Surgical technique: MIV		√	√	√				√	√					
Postoperative phase														
Chest drain management	√	√	√	√			√	√	√		√	√	√	√
Urinary drainage	√	√	√	√				√	√				√	
Early mobilisation	√	√	√	√		√	√	√	√	√	√	√	√	√

Meta-analyses

The four included meta-analyses comprised studies, which largely pre-dated the 2019 ERATS guidelines. Li et al. in 2017 reported that the range of ERAS protocol recommendations implemented was 5-11 in their included studies, with the most frequently implemented recommendations being preoperative education and early ambulation [5]. Fiore et al. in 2016 described a total of 15 ERAS elements; within their reviewed literature, the number of elements implemented ranged from 4 to 10 with a median of 6.54. The most commonly implemented elements in their report were preoperative education, prophylactic antibiotics, standardised chest drain management, and early mobilisation [4]. Li et al., 2021 reported a range of 0-22 elements with the most common being preoperative education, early ambulation, minimally invasive surgical technique, standardised chest drain management, and standardised anaesthetic protocols [7]. Zhang et al., 2021 did not comment on the specifics of ERAS protocols used in each study [8].

Primary outcome: overall morbidity

Meta-Analyses

Li et al. published a meta-analysis of seven randomised controlled trials (RCTs) with a total of 486 patients in 2017 [5]. The authors found that the rate of overall morbidity was reduced by the implementation of ERAS, with a relative risk (RR) of 0.64 (95% confidence interval (CI) 0.51-0.80, i²=1.9%) [5]. This reduction was driven by a decrease in pulmonary complications (RR 0.43, 95% CI 0.31-0.60, i²=0%) [5]. This finding was not replicated by the earlier systematic review by Fiore et al., which included one RCT and five non-randomised studies with a total of 1,612 patients; the authors found that the implementation of ERAS had no impact on the incidence of post-operative morbidity [4].

Li et al. published a further meta-analysis in 2021, including 21 articles (19 non-RCT and 2 RCT) with a total of 6,480 patients [7]. The overall complication rate was reported in 18 of the 21 studies [7]. The implementation of ERAS was associated with a reduced incidence in overall post-operative complications with a pooled relative risk of 0.64 (95% CI 0.52-0.78, p<0.001) [7]. However, there was significant heterogeneity within the pooled study data, with an i² of 63% [7].

Zheng et al. published a meta-analysis of 23 studies (11 RCT and 12 non-RCT) in 2021, with a total of 8,094 patients included [8]. The authors concluded that the introduction of ERAS was associated with a reduction in the incidence of postoperative complications (22 out of 23 studies reporting relevant data) with an odds ratio of 0.48 (95% CI 0.37-0.61, p<0.01) [8]. Once again, however, they identified significant heterogeneity within the pooled data (i²=62%) [8].

Observational Studies

There is significant variation in the included observational trials regarding sample size (range of 83 to 10,021 patients), hypothesis (ERAS vs historical control; impact of compliance with ERAS and outcomes), and design (prospective with matched historical controls, entirely retrospective). Of the included 26 observational trials, 21 reported the effect of ERAS on overall complications, with seven studies demonstrating that implementation of an ERAS protocol led to reduced overall post-operative morbidity [3,6,9-12,14,15,18-21,23,25-27,28,29,30-32]. Five of these studies were comparing the ERAS protocol to historical controls [3,9,10,11,31]. The other two studies demonstrated that improved compliance with ERAS protocols resulted in reduced rates of postoperative complications [6,12]. 

Secondary outcome: length of stay (LOS)

Meta-Analyses

All four of the included meta-analyses commented on the impact of ERAS on LOS, with all demonstrating an overall reduction in LOS either based on pooled analysis or on qualitative grounds, according to the included studies. However, the quality of the pooled analyses was generally poor due to significant heterogeneity among the included studies. Li et al. did not perform a pooled analysis due to significant heterogeneity (I² = 86.5%); among their seven included studies, four demonstrated that ERAS led to reduced LOS, while three showed no difference [5]. Fiore et al. found that LOS was significantly reduced in the included non-randomised trials, but not in the one included randomised controlled trial [4].

Li et al. divided their analysis for LOS into overall LOS (11 studies) and postoperative LOS (12 studies) [7]. Most publications showed that the implementation of ERAS was associated with reduced postoperative and overall LOS. Pooled analysis was performed for seven of the studies, with patients in the ERAS cohort having a significantly shorter LOS. This analysis had the important limitation of significant heterogeneity (I² = 98%). Zhang et al. reported a pooled analysis of 20 studies examining LOS, finding that LOS was reduced in the included ERAS patients by 2.7 days (p < 0.01). Again, this pooled analysis was subject to significant heterogeneity (I² = 97%) [7].

Observational Studies

Of the included 26 observational studies, 25 reported on the impact of ERAS on LOS [3,9-30]. Twenty of these studies found that ERAS was associated with reduced LOS after thoracic surgery [9-11,13-22,24-26,28-30-32]. The largest study included was by Alwarti et al., which encompassed a total of 10,021 patients [13]. This study examined three epochs of time: pre-ERAS (2005 to 2011), a transition period (2012 to 2015), and post-ERAS (2016 to 2019) [13]. Alwarti et al. found that the mean LOS progressively reduced from a pre-ERAS mean of 8.1 days to a transitional LOS of 7.1 days, down to an ERAS LOS of 6.6 days (p < 0.01) [13]. Similar findings were reported by another large retrospective cohort published by Van Haren et al. in 2018; this study included 2,886 patients divided into three cohorts between 2006 and 2016 [32]. This included a pre-ERAS cohort (n = 1,615), a transitional period (n = 929), and the ERAS cohort (n = 342) [32]. They reported a reduction in mean LOS from 5 days (pre-ERAS) to 4 days (transitional) to 4 days (ERAS, p < 0.001) [32]. Wang et al. reported another large retrospective cohort study including 1,749 patients [16]. This cohort comprised patients who underwent anatomical resection for non-small cell lung cancer with ERAS protocols (2016 to 2017) compared to those who had comparable surgery under the previous standard of care [16]. They found the mean postoperative LOS was reduced from six days to four days following the introduction of ERAS (p < 0.001) [16].

Secondary outcome: mortality

Due to the low event rate for perioperative mortality after thoracic surgery and the small sample size of the majority of the included studies, it is difficult to conclude the impact of ERAS on in-hospital or 30-day mortality. 

Meta-Analyses

All four meta-analyses commented on in-hospital or perioperative mortality, with no trial showing a difference between patients treated under ERAS and those without [4,5,7,8]. Zhang et al. reported an overall low event rate, with an odds ratio of 1.15 (95% CI 0.6 to 2.22, p>0.05) [8].

Observational Trials

Of the 26 included studies, 14 reported on mortality [12,13,16,18,19,22,23,26,27,29,30-32]. The only study to report a significant difference in mortality was the study by Alwarti et al., a retrospective cohort study published in 2021 [13]. This large study, which included 10,021 patients, encompassed all patients who underwent open lobectomy between 2005 and 2019, divided into three temporal cohorts as described previously [13]. The overall mortality rate for the study was 2.2% [13]. The authors reported a decreasing trend in mortality over time: from 2.8% in the pre-ERAS period to 2% during the transitional period, and finally to 1.9% in the ERAS cohort, with a p-value of 0.05 [13]. While the introduction of ERAS may have contributed to this improvement in mortality, the analysis clearly contains significant confounding factors, with multiple other potential contributors to the downtrending mortality over a 15-year period.

Secondary outcome: pulmonary complications

Meta-Analyses

Of the four meta-analyses, three reported on the effect of ERAS protocols with respect to postoperative pulmonary complications [4,5,7]. The subgroup analysis performed by Li et al. demonstrated that patients undergoing thoracic surgery under ERAS protocols had a reduced incidence of postoperative pulmonary complications, with a relative risk of 0.43 (95% CI 0.31-0.60) [5]. In the study by Fiore et al., no pooled analysis was performed; however, the included RCT demonstrated a reduction in postoperative pulmonary complications [4]. Finally, Li et al. also reported a reduction in postoperative pulmonary complications associated with ERAS, with a relative risk of 0.58 (95% CI 0.45-0.75, p<0.001) [7].

Observational Trials:

Of the 26 included studies, 13 reported on the effect of implanting ERAS on postoperative pulmonary complications [10,12,13,16,17,19,21,23,26-28,31,32]. The results were mixed, with six studies showing a reduction in postoperative pulmonary complications with ERAS and seven studies showing no difference. 

Discussion

ERAS, or more specifically ERATS (after the publication by the ERAS society in conjunction with ESTS in 2019), will play an increasingly important role in the care of patients undergoing thoracic surgery. It provides a framework for a standardised pathway of care, to streamline processes, and improve perioperative outcomes. This standardised approach to patient care is importantly multidisciplinary beginning preoperatively with adequate education and risk factor modification [3].

The most consistent finding from this systematic review was that the implementation of ERAS protocols for thoracic surgery is associated with reduced hospital length of stay. Most studies show that this reduction in length of stay is not accompanied by an increased rate of readmission. Not only is this important from a health economic perspective, but it is also vital from a patient recovery perspective [3]. Expedited postoperative recovery, with a faster return to a ‘new’ baseline function, gives a patient the best possible chance to receive additional oncological treatment such as chemoimmunotherapy [33]. Paci et al. published a study in 2017 looking at the economic impact of the implementation of ERAS for patients undergoing elective lung surgery [34]. They found that patients undergoing surgery with ERAS protocols had a reduced length of stay (four vs six days, p < 0.01) with no increase in the rate of readmission [34]. Interestingly, they also evaluated the caregiver burden on discharge, finding that patients who underwent their surgery with ERAS protocols had a trend toward a lower caregiver burden (53 +/- 90 hours vs 101 +/- 252 hours, p = 0.17) [34]. Economic costs to the healthcare system were also reduced with ERAS, with a mean difference of -$4,396 Canadian dollars [34].

Three of the four meta-analyses included reported a reduction in postoperative morbidity with ERAS. This outcome was also mirrored in seven of the 26 observational trials. It is known that postoperative complications have significant deleterious effects on patients and the healthcare system. Shinohara et al. in 2019 published a retrospective study of 345 patients who underwent lobectomy for NSCLC [35]. They found that patients who had postoperative complications, in particular pulmonary complications, had reduced five-year overall and disease-free survival [35]. Postoperative complications can also delay the time to receiving adjuvant therapy or reduce the ability of patients to tolerate adjuvant therapy. Nelson et al. in 2019 demonstrated that the implementation of ERAS for patients with clinical stage I or II NSCLC without induction therapy resulted in not only a shorter time to commencing adjuvant therapy but importantly increased rates of receiving adjuvant therapy (40% vs 62%, p<0.001) [36].

A significant limitation of any systematic review is the quality of evidence provided by the included studies. Most of the evidence comes from meta-analyses of small randomised controlled trials and observational trials and from individual prospective and retrospective observational trials with varying sample sizes. Furthermore, there is significant heterogeneity in the design of ERAS protocols between the reported studies, and the compliance with each individual component of the protocol within each study. From Rogers et al., 2018, and Forster et al., 2020, improved compliance with ERAS protocols leads to improved outcomes with lower postoperative morbidity and shorter lengths of stay. There is not yet sufficient evidence with which to comment specifically on the impact of the recently released ERATS guidelines; this will be an important avenue for further research [1]. One benefit of a more standardised approach to ERAS in thoracic surgical patients is greater comparability of findings between studies, in addition to greater transferability of results across patient populations and centres.

## Conclusions

Overall, ERAS/ERATS is an important adjunct to the management of patients requiring thoracic surgery, consistently leading to shorter lengths of stay and likely contributing to reduced rates of postoperative morbidity. Further research will be required to determine the impact of the recently released ERATS guidelines as these are more comprehensive with forty-five total elements. Furthermore, it will be important to critically analyse each of the forty-five elements to ensure that they are effective without becoming cumbersome, thereby increasing uptake across the board.

The importance of ERAS/ERATS in shortening the length of stay and potentially lowering postoperative morbidity will continue to become apparent as patients become older, more comorbid, and healthcare resources become more constrained. By consistently reducing the length of stay without increasing the rate of readmission, the application of ERATS/ERAS may contribute to a more efficient use of scarce healthcare resources. Furthermore, by reducing postoperative morbidity, it may improve access to adjuvant onco-immunotherapy, further improving overall- and disease-free survival. It will be vital, therefore, that thoracic surgery encompasses a multidisciplinary, guideline-based approach to streamline patient care as much as possible, with a view to optimising outcomes.
